# Health-related quality of life of children with X-linked hypophosphatemia in Germany

**DOI:** 10.1007/s00467-024-06427-0

**Published:** 2024-06-25

**Authors:** Martin Klein, Michael Obermaier, Helena Mutze, Sophia Maria Wilden, Mirko Rehberg, Karl Peter Schlingmann, Dorothee Schmidt, Oliver Metzing, Angela Hübner, Anette Richter-Unruh, Markus J. Kemper, Marcus Weitz, Elke Wühl, Norbert Jorch, Ludwig Patzer, Clemens Freiberg, Sabine Heger, Miroslav Ziviknjak, Dirk Schnabel, Dieter Haffner, on behalf of the German Society for Pediatric Nephrology (GPN) and the German Society for Pediatric and Adolescent Endocrinology and Diabetology (DGPAED)

**Affiliations:** 1 Department of Social Sciences, Catholic University of Applied Sciences North Rhine, Westphalia, Cologne, Germany; 2 Department of Pediatrics, Faculty of Medicine and University Hospital Cologne, University of Cologne, Cologne, Cologne, Germany; 3 Department of General Pediatrics, Pediatric Nephrology, University Children’s Hospital, Münster, Germany; 4 Division of Pediatric Endocrinology and Diabetes, Department of Pediatrics and Adolescent Medicine, University of Lübeck, Lübeck, Germany; 5 University Children’s Hospital, Jena, Germany; 6 Division of Pediatric Endocrinology, Department of Pediatrics, Medizinische Fakultät Carl Gustav Carus, Technische Universität Dresden, Dresden, Germany; 7 University Children’s Hospital Bochum, Bochum, Germany; 8 Asklepios Children’s Hospital Hamburg-Heidberg, Hamburg, Germany; 9 Department of Pediatrics I, University Children’s Hospital Heidelberg, Heidelberg, Germany; 10 Division of Pediatric Nephrology, Center for Pediatrics and Adolescent Medicine, Heidelberg University, Heidelberg, Germany; 11 University Children’s Hospital, Evangelisches Klinikum Bethel, Bielefeld, Germany; 12 St. Elisabeth and St. Barbara Children’s Hospital, Halle/Saale, Germany; 13 Department of Pediatrics and Adolescent Medicine, University Medical Center Göttingen, Göttingen, Germany; 14 Kinderkrankenhaus auf der Bult, Hannover, Germany; 15 Department of Pediatric Kidney, Liver and Metabolic Diseases, Hannover Medical School, Carl-Neuberg-Str. 1, D-30625 Hannover, Germany; 16 Center for Chronically Sick Children, Pediatric Endocrinology, University Medicine, Charitè, Berlin, Germany

**Keywords:** X-linked hypophosphatemia, Rare diseases, Health-related quality of life, Children, Adolescents, Questionnaire KIDSCREEN-52

## Abstract

**Background:**

X-linked hypophosphatemia (XLH) is a rare inherited phosphate-wasting disorder associated with bone and dental complications. Health-related quality of life (HRQoL) is reduced in XLH patients on conventional treatment with phosphate supplements and active vitamin D, while information on patients treated with burosumab is rare.

**Methods:**

HRQoL was assessed in 63 pediatric XLH patients participating in a prospective, observational study and patient registry in Germany using the KIDSCREEN-52 survey instrument and standardized qualitative interviews.

**Results:**

The median age of the XLH patients was 13.2 years (interquartile range 10.6 – 14.6). At the time of the survey, 55 (87%) patients received burosumab and 8 (13%) conventional treatment. Forty-six patients (84%) currently being treated with burosumab previously received conventional treatment. Overall, HRQoL was average compared to German reference values (mean ± SD: self-report, 53.36 ± 6.47; caregivers’ proxy, 51.33 ± 7.15) and even slightly above average in some dimensions, including physical, mental, and social well-being. In general, XLH patients rated their own HRQoL higher than their caregivers. In qualitative interviews, patients and caregivers reported that, compared with conventional therapy, treatment with burosumab reduced stress, bone pain, and fatigue, improved physical health, and increased social acceptance by peers and the school environment.

**Conclusions:**

In this real-world study in pediatric XLH patients, HRQoL was average or even slightly above that of the general population, likely due to the fact that the vast majority of patients had their treatment modality switched from conventional treatment to burosumab resulting in improved physical health and well-being.

**Graphical abstract:**

**Figure:**
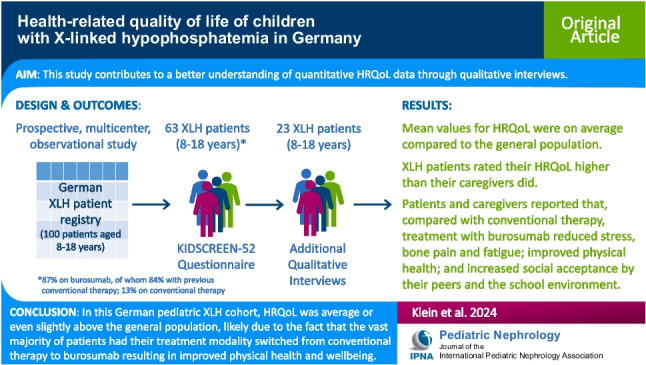
A higher resolution version of the Graphical abstract is available as Supplementary information

**Supplementary Information:**

The online version contains supplementary material available at 10.1007/s00467-024-06427-0.

## Introduction

X-linked hypophosphatemia (XLH), is a rare, inherited, phosphate-wasting disorder with a prevalence of 4–5 per 100,000 children [1–3]. It is caused by pathogenic variants in the *PHEX* (phosphate-regulating neutral endopeptidase homolog X-linked) gene which is mainly expressed in the bone and teeth, resulting in elevated circulating levels of the phosphaturic hormone fibroblast growth factor 23 (FGF23) causing renal phosphate wasting and impaired synthesis of 1,25-dihydroxyvitamin D (1,25(OH)_2_D) and consecutive hypophosphatemia. The latter causes rickets and osteomalacia with the clinical consequences of disproportionate short stature, skeletal pain, delayed walking, bone pain, and lower-limb deformities in children. In addition, patients may develop craniosynostosis and are prone to muscle weakness as well as dental abscesses [4]. Adults with XLH show additional features, including pseudofractures, enthesopathies, osteoarthritis, and hearing loss.

Conventional treatment with oral phosphate supplements and active vitamin D only partly ameliorates XLH-associated complications and is associated with significant side effects resulting in substantially impaired health-related quality of life (HRQoL) in these patients [5–10]. Maintenance and restoration of appropriate HRQoL are essential for a successful patient treatment process, as well as for managing the challenges of everyday life, as medical interventions will not merely maintain life or restore a certain level of health, but also bear consequences that may affect the reality of life of XLH patients (i.e., adverse effects).

Recently, burosumab, a fully human anti-FGF23 monoclonal antibody, has become available for treatment of pediatric and adult XLH patients in Europe, the USA, and Canada [11]. Burosumab was shown in a randomized controlled trial to be substantially more effective compared to conventional treatment in children with XLH with respect to healing of rickets, and improvement of lower limb deformities and mobility and not associated with severe side effects [12]. In a randomized clinical trial comparing burosumab with conventional treatment in 35 children with XLH, patient-reported outcomes (PROs) at weeks 40 and 64, including pain interference and fatigue scores, significantly improved with burosumab but not with conventional therapy compared to baseline [13]. Real-world data on HRQoL of children and adolescents with XLH in the era of burosumab are not available. To this end, we investigated HRQoL of pediatric XLH patients enrolled in a German prospective, multicenter, observational study and patient registry, the vast majority of whom are treated with burosumab, using KIDSCREEN-52 questionnaires and qualitative interviews.

## Methods

This is an analysis of the prospective, multicenter, observational cohort study and patient registry “Growth and comorbidity in children with X-linked hypophosphatemic rickets” of the German Society for Pediatric Nephrology (GPN) and the German Society for Pediatric and Adolescent Endocrinology and Diabetology (DGPAED), initiated in 2018 [14]. The study/registry received appropriate ethics committee approval from the Institutional Ethics Review Board at Hannover Medical School (No. 7259) and from each participating center and was performed in accordance with the Declaration of Helsinki. Written informed consent was obtained from all parents/guardians, with age-appropriate consent or assent from patients.

In this study/registry, children and adolescents were eligible if diagnosed with XLH based on family history and/or genetic confirmation, presence of clinical and/or radiological signs of rickets, impaired growth velocity and serum phosphate levels below the age-related reference range, associated with selective renal phosphate wasting, i.e., reduced age-related maximal rate of tubular phosphate reabsorption divided by the glomerular filtration rate (TmP/GFR) using a 2nd morning spot urine and parallel blood sampling, in the absence of vitamin D or calcium deficiency [2, 4]. A total of 134 pediatric XLH patients aged between 0.1 and 18 years were enrolled in Germany between November 2018 and May 2023. One hundred of these patients were aged between 8 and 18 years which was the inclusion criteria for the study on HRQoL. The KIDSCREEN questionnaires were developed on a sample of children and adolescents at this age. The items were developed for this target group; normal values are offered and the T-values were calculated. It has been shown that younger children do not understand some questions or that some questions are inappropriate in this age group. In total, 63 children and adolescents agreed to participate in the HRQoL survey and 23 of those in qualitative interviews, respectively. Due to the interdisciplinary approach, a paper-based survey (KIDSCREEN-52) was used and supplemented with guided telephone interviews with children and adolescents or their caregivers. This allowed a deeper insight into this complex matter. With this approach, we respect the bio-psycho-social model of the International Classification of Functioning, Disability and Health (ICF). The ICF of the World Health Organization (WHO) is intended to enable internationally uniform communication on the effects of health problems, taking into account the entire life background of a person [15]. The ICD-based XLH diagnosis was expanded with additional information on contextual factors so that a broader and more appropriate picture of the HRQoL of the children and adolescents with XLH is possible. The bio-psycho-social model considers functional impairment in interaction with contextual factors. The contextual factors include both environmental and personal factors. The contextual factors are often influenced by the social environment. Quality of life is thus not primarily recognized as a person’s medical problem, but as a socially determined problem [16].

### KIDSCREEN-52

The standardized questionnaires KIDSCREEN-52 (child and adolescent version 8 to 18 years for Germany) and the KIDSCREEN-52 (parents’ version for Germany) comprises 52 questions in ten dimensions: Physical Well-Being (5 items), Psychological Well-Being (6 items), Moods and Emotions (7 items), Self-Perception (5 items), Autonomy (5 items), Parent Relations and Home Life (6 items), Financial Resources (3 items), Peers and Social Support (6 items), School Environment (6 items), and Bullying (3 items). KIDSCREEN-52 has five possible answers for each dimension, scored from 1 to 5 [17].

All questionnaires were anonymous. Due to the usual low number of cases of rare diseases, datasets with only one missing item were also included in the analysis. Eight KIDSCREEN-52 questionnaires (3 Self-report/5 Proxy) had more than one missing item, so they could not be used for the evaluation. The KIDSCREEN-52 survey tool continues to be a usable and valid survey tool because socio-demographic correlations as well as judgment effects are consistent. Age- and gender-specific norm scores could be determined using a large and nationally representative sample [18]. Thus, very comprehensive information on psychometric properties in classical and probabilistic test theory is available for both the KIDSCREEN-27 (27 questions) and KIDSCREEN-10 (10 questions) indices in self and parental judgment. Both procedures are widespread and proven, and allow for a fair comparison of HRQoL in children and adolescents from different European countries because of their internationally conducted validation and standardization [18–20]. KIDSCREEN is validated and used in studies worldwide. Most articles are published in Spain, followed by Portugal. Overall, more articles are published in Europe than in the rest of the world. In South America, Asia, Africa, North America, Oceania, and Central America, a total of 60 publications have been published [21].

All XLH patients included in this registry were asked by their treating physician to complete the questionnaires and participate later on in the standardized telephone interviews when they visited the outpatient clinic. They had the opportunity to decide for themselves whether or not they wanted to complete the questionnaire and participate in the telephone interviews.

### Qualitative interviews

Based on the dimensions of the KIDSCREEN-52 questionnaire, an open interview guideline with in-depth questions was developed, which is based on a multidimensional construct of HRQoL. The structured interview was designed to prevent inconsistency in responses. The questions are standardized and were asked by the same specially trained research assistant to reduce variability in responses. There is a standardized guide to reduce subjectivity in the interpretation of questions. To ensure construct validity, the questions were structured using the ten predetermined items from the internationally recognized and validated KIDSCREEN questionnaire. To ensure content validity, we also conducted expert interviews. After extensive literature research, expert interviews, and a pretest, two items were removed. Questions on Parent Relations and Home Life and Financial Resources were felt to be too personal for a telephone interview. The interviewees’ feedback on the comprehensibility of the structured interview was positive. After obtaining informed consent, implementation took place in the form of telephone interviews of approximately 1 h in duration, which were recorded as audio files and then transcribed. In the course of the proxy interviews, representatives were always reminded to take the perspective of the interviewee as much as possible, taking into account individual experiences. As the state of research on the HRQoL of patients with XLH is rudimentary and, accordingly, few robust hypotheses can be used, the principle of openness represents an essential approach in this procedure. In this respect, the subjective perspective of those affected takes on a central role. During the interview situation, a comprehensive overview of the reality of life with XLH is made possible through individual assessments. Furthermore, the interview situation offers the possibility of clarifying misunderstandings and allows a more precise understanding of complex and individual topics such as HRQoL. For example, the subjective significance of the XLH condition for the assessment of quality of life can be determined and other relevant factors can be collected. Furthermore, additional research-guiding hypotheses can be generated. This mixed-methods design enables a comprehensive, both comparable and individual, assessment of the HRQoL of the patient group.

### Statistical analysis

The KIDSCREEN Group Europe recommends two methods to analyze the obtained data. One is to evaluate and analyze the data through SPSS statistical and analysis software [17]. This method was used for this survey. The first step was to create the “Data mask” and to enter the data from each of the last surveys. This step was done in close accordance with the English Data mask as well as the manual [17]. In the next step, data were summarized along the ten dimensions and *T*-values were generated using the KIDSCREEN-52 syntax. This allowed for international referencing of the results. The values are based on data from 12 European countries. Following the recommendations of the KIDSCREEN Group European data will be compared between group scores on the KIDSCREEN scales and the reference population [17]. For this purpose, the mean values and standard deviations of the dimensions are used (Table 1) as well as the *T*-values, which are compared with international and German *T*-values. A qualitative content analysis was carried out using MAXQDA software. The individual interview files were given predefined codes based on the question items of the interview guide. With the help of the codes, the content allocation of the statements and the analysis of the interviews were implemented. Data is presented as mean ± SD or median (IQR) according to Shapiro-Wilk normality tests. All statistical analyses were performed with IBM SPSS Statistics version 29.

**Table 1:** *T*-values of health-related quality of life in 63 pediatric XLH patients enrolled in this registry rated by patients and caregivers using the KIDSCREEN-52 questionnaire

Scale		Self-report 8–18								Proxy 8–18							
		*N*	Mean	SD	Percentiles					*n*	Mean	SD	Percentiles				
					10	25	50	75	90				10	25	50	75	90
Physical	T_i	21087	50.00	10.00	38.47	42.53	49.63	55.60	64.30	15696	49.98	10.01	36.70	43.66	49.54	55.89	63.68
	T_g	1692	52.36	8.73	42.47	47.02	52.43	58.93	64.30	1661	51.45	8.79	39.32	46.28	52.64	56.94	63.65
	T_p	52	52.46	10.95						56	48.40	9.82					
Psychological Well-being	T_i	21311	50.00	10.00	36.91	43.25	49.34	57.60	61.55	15777	49.99	10.00	36.88	43.47	48.87	58.18	61.09
	T_g	1696	52.80	9.00	41.54	47.13	52.29	57.60	68.49	1689	51.56	8.68	40.73	46.59	52.12	58.18	61.11
	T_p	55	51.21	8.37						56	52.50	9.37					
Moods and Emotions	T_i	21208	50.00	10.00	37.76	42.50	49.09	57.40	62.06	15723	49.99	9.99	37.97	43.87	48.57	58.00	62.68
	T_g	1707	51.10	9.93	39.32	43.91	49.66	57.40	65.33	1689	49.07	9.94	36.61	41.93	48.57	54.64	62.68
	T_p	55	52.04	11.33						55	49.18	12					
Self-Perception	T_i	21306	50.00	10.00	39.21	43.17	47.78	55.38	69.78	15816	49.99	10.00	38.88	42.28	49.11	56.18	61.43
	T_g	1700	51.51	9.99	40.02	44.45	49.76	58.96	69.78	1690	51.07	9.64	39.64	44.25	49.11	56.18	62.91
	T_p	55	54.90	10.78						56	49.74	12.38					
Autonomy	T_i	21326	50.00	10.00	37.35	43.59	48.70	56.27	68.75	15897	50.01	10.01	37.60	43.48	48.22	57.07	67.95
	T_g	1704	53.25	8.59	43.33	48.37	53.20	58.34	68.75	1693	54.59	8.22	45.67	48.22	53.87	61.01	67.95
	T_p	54	52.76	9.86						56	52.23	8.19					
Parent Relations and Home Life	T_i	21148	50.00	10.00	36.98	42.55	49.50	58.53	65.87	15709	50.00	10.01	38.16	42.33	49.38	58.45	62.45
	T_g	1687	50.28	9.04	39.46	44.46	49.50	55.67	65.86	1672	49.72	8.62	38.18	44.46	49.38	55.13	61.61
	T_p	55	54.83	8.76						56	52.00	9.40					
Financial Resources	T_i	21006	50.00	10.00	37.47	41.92	49.28	56.35	62.86	15595	50.00	10.00	35.23	43.31	51.90	59.33	65.02
	T_g	1675	53.63	8.90	41.92	49.10	54.47	62.86	62.86	1689	55.27	7.55	46.03	51.76	55.39	61.81	65.02
	T_p	54	56.14	8.53						56	59.00	8.21					
Social Support and Peers	T_i	21130	50.00	10.00	38.15	43.60	48.35	54.93	62.66	15485	49.99	10.01	38.60	44.42	50.73	55.44	63.16
	T_g	1701	50.55	9.11	40.39	45.07	50.24	54.94	62.65	1666	50.73	8.57	40.45	46.40	50.74	55.44	60.38
	T_p	54	53.07	10.20						56	50.04	10.48					
School Environment	T_i	21051	50.00	10.00	38.15	43.82	48.61	56.40	61.87	15697	49.99	10.00	37.40	43.31	49.75	57.01	62.47
	T_g	1675	51.55	9.58	40.15	45.30	50.44	56.66	64.90	1685	50.73	9.25	39.11	45.27	50.32	56.88	62.48
	T_p	54	54.62	10.43						55	52.20	10.68					
Social Acceptance	T_i	21318	50.00	10.00	35.44	42.20	48.07	58.85	58.85	15871	50.00	9.99	34.63	44.83	50.55	58.83	58.83
	T_g	1685	50.03	9.74	35.54	42.20	48.99	58.85	58.85	1695	49.92	10.09	34.78	44.83	50.55	58.83	58.83
	T_p	54	52.21	8.98						55	48.26	12.78					

*T_i*, *T*-values international; *T_g*, T-values Germany; *T_p*, *T*-values patients/proxy

## Results

The study included 63 children and adolescents with XLH (36 females) aged between 8 and 18 years. Their median age was 13.2 years (interquartile range (IQR) 10.6 – 14.6). Patients were diagnosed with XLH at a median age of 0.8 years (IQR 0.0 – 2.9). There was a positive family history for XLH in 37 out of 63 patients (59%). The diagnosis was confirmed by genetic testing in 82% of patients including all patients with a negative family history of XLH. In 48 cases, both caregivers and patients completed the KIDSCREEN-52 questionnaires. In eight cases only the patients and in seven cases only the caregivers completed the forms. At the time of the survey, 55 (87%) patients received burosumab and 8 (13%) conventional therapy. The median age at initiation of burosumab therapy was 10.9 years (IQR 9.1 – 12.2); 46/55 (84%) patients received prior conventional treatment, initiated at a median age of 2.4 years (IQR 0.9 – 4.2), over a median period of 8.6 years (IQR 5.1 – 10.5). Complementary qualitative interviews were conducted with 23 subjects (6 patients and 17 caregivers) targeting group-specific parameters (medication, forms of psychosocial support, differentiated views/perspective on HRQoL) to obtain reliable explanations. These interviews provided important insights into the change in HRQoL in XLH patients enrolled in this registry who were switched from conventional therapy to burosumab. Differences between children (age 8–11 years) and adolescents (age 12–18 years) were evident, as were differences in caregiver assessments.

### KIDSCREEN-52 questionnaires and qualitative interviews

The means (± SD) of all *T*-values of HRQoL were 53.36 ± 6.47 and 51.33 ± 7.15 in the children’s and adolescents’ self-report questionnaires (*n* = 55) and caregivers’ proxy questionnaires (*n* = 56), respectively. Thus, the mean values for HRQoL in our patient cohort were slightly higher than the mean values in the general population of 50 (Table 1). Interestingly, the mean scores on the proxy questionnaires were generally lower than the self-report questionnaires, suggesting that patients aged 8 to 18 years rated their own HRQoL higher than their caregivers did. This was especially true for the dimensions “School Environment” and “Social Acceptance.” In our dataset, these were equal to or slightly above average in three out of four cases. Caregivers reported only a mean *T*-score of 48.26 ± 12.78 for the dimension “Social Acceptance,” which is just below the mean for the general population. The self-assessments are better (52.21 ± 8.98).

The mean *T*-scores of caregivers (52.2 ± 10.68) and self-assessments (54.62 ± 10.43) of the dimension “School Environment” are above the *T*-scores of the general population. This is also confirmed in the dimension “Social Support and Peers.” The mean *T*-scores of self-reports (53.07 ± 10.43) are also higher than those of caregivers (50.04 ± 10.68). Almost all qualitative interviews (21/22) show that the monthly burosumab injections reduce the treatment effort, especially for the dimensions “Social Acceptance” and “School Environment.” This means that taking the medication does not play a major role in the lives of children and adolescents compared to previous treatment: “And that is a total improvement in the quality of life with this injection. Just not having to think about it anymore and not having to take a pill in front of other kids. That’s just noticeable and that's a difference like night and day” (002_005(1), 16.29; Table 2).

**Table 2:** Results of health-related quality of life assessed by qualitative interviews

Dimension	Qualitative interviews—quotation
Physical well-being	“The difficult thing was to take the medication every three hours [...] Yes, with an alarm clock. So that restricted us, that has to be said. During the day, you got used to it, every three hours. But it always had to be organised when she was playing somewhere, when we were away somewhere. We had to make sure that it worked when she was still very small. Then at some point we got a clock that beeped, so she remembered when she played. That worked well, actually. Yes, in the evenings and at night it was very exhausting. I think for [name] it wasn't that bad, but for my husband and me it was bad” (004_008(1), 10.26) "It has just become clear, the stress has just gone away. When you're still being reminded by your teachers in primary school that you have to take your medicine, that was all still possible. But when you're at high school and you have to remember to take your medicine six times a day during class, then you're already being looked at. And now it's like this, I come home from school, inject myself in the evening or my mother comes home from work, gives us the injection and then everything is ready again. If we're not at home, we can order it to be there a day earlier. It doesn't matter then. It's much easier" (004_001(1), 14.33) "Yes, we think that this new medication is incredibly good. So of course, we don't know yet what will be in many years or what will come, but at this point in time, we have the impression that she’s growing tremendously. It has come at exactly the right time. Nobody would think that she has something or could have something, because she is exactly, well, the medication is taken in her noticeable growth phase and she is now in puberty and we are happy about every day that it continues properly. [...] It can still go on properly. Straight growth and I would say straightening of the musculoskeletal system due to burosumab" (002_002(1), 21.31) “I didn't even think about the abdominal pain […]. The [Off-label-medicine] was always a big problem, because it works like a laxative and then there were always these cramps. That was also unfortunate sometimes, when she grew a lot in primary school and then the dose was increased and then it was always extremely associated with diarrhoea. And that was also very unpleasant for her, even at school. So that we often let her stay at home when it was like that" (004_008(1), 59.33)
Psychological well-being	"But her psyche has suffered so much over the years [...] so at 13, 14, 15 years of age. Because then it just got worse and worse with her legs" (010_001(1), 15.45) "It takes a lot of time, all the doctor's appointments, and it always has to do with stress” (04_01(1), 04.30) "And to be happy. That is also important for him. To be happy and that he doesn't have so many health problems. That worries him from time to time" (001_001(1), 02.16)
Moods and Emotions	"I do think that it puts a strain on her, that is, her body size. Sometimes she gets along quite well, but then she also gets teased about her height. But just in a nice way, not in a nasty way. That's how it is among friends. And there are also phases where she is disappointed, or yes, that’s how I would put it" (04_08(1), 37.45) "I think she herself doesn't see it as so bad now. Well, she can't reach everywhere, but for her it's not a restriction. It's just that all the other people seem to have a problem. And that makes it her problem from a psychological point of view” (002_003(1), 01.27.21) "And I will also say that she is not well and that she is completely isolated here and does not go out because she is also embarrassed, she has such a shoe. So, her crooked leg is just too short and then she has to wear a shoe with a thick sole" (010_001(1), 55.25)
Self-Perception	"Well, she feels good in her body. Whereas, if she could change that, she would probably want to straighten her legs. Yes. [...] But she wears everything she likes. Even if it's a short pair of shorts or leggings. She wears everything. She thinks it looks good. That's what she wears. And she doesn't care if the others look. She thinks it looks pretty and then she wears it with pride. And I think that's great" (04_03(1), 32.50) "[...] She actually questions it. So 'Why do I actually have this? And others don't? So, it's all still within reason. But there’s a little bit where I notice, okay, she perceives it differently than she did two/three years ago" (04_04(1), 39.24) "Personally, I wouldn't use the term [disease] like that. I think I would also explain it like that later, when she can have a little understanding of how a body is formed. Then I would say that some part is broken and we repair it with this injection and everything is normal for them. And the term disease doesn't have to come up at all" (032_002(1), 27.13)
Autonomy	The improvement in quality of life is essential, so he doesn't always have to look at his mobile phone or watch the clock. He knows, 'Every two weeks I get the injection' and that's it for him. So that's actually ideal. He is completely independent. If you always have subliminal appointments, yes, and you are a child or a teenager, then that already interferes with normal everyday life. And that has completely disappeared. He is much more relaxed about it now" (001_001(1), 15.09) "Even in kindergarten they have always moved around a lot, even outside of kindergarten, he was the only one allowed to take his running bike. So that he could keep up. So, he learned to ride a running bike very quickly and even when we went on trips, we always had his running bike with us and so he was always on the move. [...] But he also knows why he can take it with him. And the other children knew that too" (003_002(1), 12.25) "That everywhere I go, I no longer have to remember that I always have this medication with me. And that I'm not always reminded every two hours by a watch or something that I have to take it" (009_003(1), 13.05) "Everyday examples now are simply sleepovers at friends' houses. We did that before, but then it was always highly complicated because we had to coordinate it with the phosphate administration. And now she can simply do it. And now, for the first time, I have asked her if she would like to go to a holiday camp. I avoided that before because I knew it would be too complicated. [...] But I asked her for the first time, I wouldn't have done that before" (004_004(2), 15.41)
Social Support and Peers	"So [he] also suffers a bit with his psyche. That he always says 'He is always the smallest'. And that the children who don't know him don't believe that he's already in the fifth grade, because he's as tall as a first grader" (032_005(1), in: 032_006(1), 41.40) "I don't know. It [XLH] is just there. [...] So apart from the medication, maybe the fact that my body doesn't exactly correspond to the Instagram ideal image and I somehow look noticeably different from others and I somehow have no stamina and no strength and so on (04_02(1), 29.35) "What you noticed at the beginning when she went to music school was that the instrument was already the smallest and yet her fingers were still too small. So these are such little things. Or now in the judo club you first have to find a child in the competition who is the same age and in the same weight category. In the beginning, [name] had to compete with five-year-olds because there was no one else. So if she had to compete with children of the same age who are two heads taller and of course heavier, that would have been unfair. So things like that, of course it stands out that they are smaller and of course also lighter" (032_006(1), 15:40)
School Environment	"Yes, yes, and the physical education teacher is also really great. She came up with wonderful things to integrate her. For example, when she is not allowed to do things like long jump. Then, for example, she is her assistant and gets a [very good grade] for the assistance. Because she then helps to measure and enter the grades and so on. And she is very happy with that. [...] Exactly, she is then involved and also gets a grade. That is important for children” (002_005(01), 33.23). "Yes, actually I participate [in school sport]. And I actually participate more than I should. Sometimes beyond my pain threshold. But I don't feel like always being the sick one " (04_02(01), 22.48) "No, there are always situations like that. For example, she was recently at the climbing park and there are size restrictions. So you're not allowed to go on all the routes if you're under that size. She's always a bit scared, I notice that too. [...] Or now they have their bicycle test in fourth grade. And then the bikes are all incredibly big. So these are already situations where she is made aware that there is a certain difference to the others. And then I just said, 'Shall we call them and ask if you can take your own bike with you? But she thinks that's stupid, too. Then she says, 'Then I'd rather stretch out or ride standing up so I can reach the pedals.' She doesn't want to have a different role there” (002_005(01), 48.02)
Social Acceptance	"And that is a total improvement in the quality of life with this injection. Just not having to think about it and not having to take the tablet in front of other children. That's just noticeable and it's a difference like night and day" (002_005(1), 16.29) "Well, but it happens more often, I have to say, that she is seen both by children at school or by adults in some contexts as very, very young. That is a bit annoying. [...] She always immediately says 'Watch this! I'm already ten years old. And sometimes when it annoys her, she is also rude. And I allow her to do that too. I mean, she shouldn't offend people, of course, but we somehow try to find a healthy balance, that she's allowed to be annoyed" (002_005(1), 31.08) "So that one person taps the other and says 'Hey, look' or some shake their head or some ask, 'What went wrong with you? And that hurts so much [...]." (010_001(01), 38.09) "But it is very important to her that we don't say anywhere, 'She has this and that and please don't rate her'. She goes all the way. We know that and that is never, never, never an issue now" (002_002(01), 10.11)

The dimension “Physical” is slightly better rated by children and adolescents than by the general population (52.46 ± 10.95 vs. 52.36 ± 8.73), while caregivers rated the physical dimension lower (48.4 ± 9.82) than the general population (48.4 ± 9.82 vs 51.45 ± 8.79). This is further supported by the qualitative interviews: “Nobody would think that she has something or could have something, because she is well, the new medication (burosumab) is taken in her noticeable growth phase and she is now in puberty and we are happy about every day that it continues properly. [...] It can still go on properly. Straight growth and I would say straightening of the musculoskeletal system due to burosumab” (002_002(1), 21.31; Table 2). For 18 out of 23 patients (78%), their HRQoL is likely to be important in terms of being less noticeable and being able to participate in social activities despite possible physical limitations. The side effects of conservative therapy such as abdominal pain, cramps, and diarrhea have disappeared. Nine out of 23 patients (39%) also reported better growth, improvement in the musculoskeletal system, reduced fatigue symptoms, and/or less bone pain (Table 2).

It is noteworthy that the self-assessment of the children and young people is only slightly below the normal values in two dimensions: “Psychological Well-being” and “Autonomy.” The dimension “Psychological Well-being” includes positive emotions and life satisfaction and reflects the children’s and adolescents’ view of their satisfaction in life to date (51.21 ± 8.37). The mean *T*-scores of the German population are higher (52.80 ± 9.00) in this dimension and it is viewed more positively by caregivers (52.50 ± 9.37). Here, the mean *T*-scores are higher than the reference values (51.56 ± 8.68). In the interviews, the caregivers also pointed out differences in psychological well-being (*n* = 20) between the time on conventional therapy and burosumab therapy. “Fewer appointments with doctors are necessary, there is a lot of research in the field of XLH, new studies appear regularly and there is hope for a normal life for the first time after many years of suffering.” But bad days and teasing are also reported in the “Moods and Emotions” dimension. However, it is also reported that due to the support in their social environment, the view is taken that the children and adolescents react with indifference on good days.

In contrast, the “Moods and Emotions” dimension shows the extent to which the child/adolescent experiences depressive moods and emotions, as well as distressing feelings such as loneliness, sadness, inadequacy/insufficiency, and resignation. This dimension shows a high score on the HRQoL when these negative feelings are rare. Self-report scores are higher than the average for the normal population (52.04 ± 11.33). However, caregivers rate this lower (49.18 ± 12). “And I will also say that she is not well and that she is completely isolated here and does not go out because she is also embarrassed, she has such a shoe. So, her crooked leg is just too short and then she has to wear a shoe underneath with a thick sole” (010_001(1), 55.25; Table 2).

A similar picture emerges in the dimension of “Self-Perception.” Here, the children/adolescents rate their satisfaction with their body image, appearance, and clothing and other personal accessories. This dimension also reflects the value of how positively others value him/her. Here, too, the ratings in the self-reports are better than from the caregivers’ (54.90 ± 10.78 vs 49.74 ± 12.38).

The dimension “Autonomy” looks at the opportunity given to children or adolescents to create their social and leisure time. It refers to the child’s/adolescent’s freedom of choice, self-sufficiency, and independence. This is the only dimension in our study that was rated below the German reference values but above international reference values by both children and adolescents (52.76 ± 9.86) and caregivers (52.23 ± 8.19).

## Discussion

In this German pediatric XLH cohort, the vast majority of whom are treated with burosumab, the overall mean HRQoL assessed by KIDSCREEN-52 questionnaires was slightly above the mean of the general population. The mean *T*-value of self-reported HRQoL was slightly below the normal values in only two dimensions, “Psychological Well-being” and “Autonomy,” while the mean values of the dimensions “Social Support and Peers,” “Parents Relations and Home Life,” “Social Support and Peers,” “School Environment,” and “Financial Resources” were even slightly above the *T*-values of the general population suggesting that their overall HRQoL can be considered to be at least average. The qualitative interviews suggest that this observation is at least partly related to the fact that burosumab treatment improved physical health and well-being and, in contrast to conventional treatment, does not play a negative role in daily life. In general, XLH patients enrolled in this registry rated their own HRQoL higher than their caregivers did. This was especially true in the dimension “School Environment,” which explores a child’s/adolescent’s perception of their cognitive capacity, learning, and concentration and their feelings about school. It also includes their satisfaction with their ability and performance at school [17]. This was also true for the dimension “Social Acceptance.” These dimensions cover the aspect of feeling rejected by peers at school and the feeling of fear of peers. The idea of inclusion is particularly emphasized. The former frequent use of medication led to stress, which also affected the “Physical Well-being.” These differences are consistent with QoL studies conducted in children with nephropathic cystinosis, children with hemophilia, and children with attention deficit hyperactivity disorder and their caregivers [22–25]. This could be because the caregivers transfer and compare their own school experiences to the children and young people. The children and adolescents do not know it any differently and therefore lack a comparison. In addition, it could be possible that in the comparison of the caregiver generation the social inclusion efforts over recent decades lead to a somewhat better understanding of disadvantage, disability, and discrimination. It could also be that the children and adolescents make a comparison before and after their change in medication. In the past, oral phosphate supplements were taken every 2 to 3 h in the presence of the others (peers), but this no longer plays a role. In addition, many teachers, as well as peers, are supportive and this often seems to lead to the children and adolescents with XLH being more confident and testing their limits despite numerous obstacles.

On comparing our results in the dimension of “Physical Well-being” with others the patients with XLH in these other studies had poorer HRQoL results [5–7, 13]. Approaches to explanation can be found in the qualitative interviews, for example in the physical dimension (Table 2). This suggests that “normal” HRQoL was at least partly related to improved physical well-being by burosumab treatment. The good HRQoL is probably at least partly related to the fact that patients treated with burosumab usually do not experience treatment-related side effects, which are generally mild and, in contrast to conventional treatment, were not reported in the qualitative interviews. Another explanation for these results could also be found in the dimension “Parent Relations and Home Life.” The quality of interaction between the child/adolescent and caregivers is higher than average for both self-reports (54.83 ± 8.76) and caregivers (52.00 ± 9.40). This indicates whether the child/adolescent feels loved and supported by the family, whether the atmosphere at home is pleasant, and whether the child/adolescent feels fairly treated.

Previous studies in children and adolescents with XLH reported a significant burden of disease, despite treatment with frequent oral phosphate supplements and active vitamin D, including bone pain and impaired physical functioning that persists throughout childhood [7]. Health-related QoL shows anxiety and depression in about 25% of children on conventional treatment and problems with walking and self-care in about 50 – 60%. In these studies their QoL is lower than that of both the general population and patients with other chronic diseases, mainly due to diagnostic delay, treatment difficulties, poor psychosocial support, and problems with social integration. Early diagnosis and optimal treatment are very important to control the disease in children and adolescents with XLH to avoid complications and maintain or improve their HRQoL [7, 13]. The new treatment modality with burosumab was approved by health authorities in Germany and other European countries only in 2018. Due to this, the daily multiple intakes of medication are still very present among patients and caregivers. It must be considered that this may still have positive effects on self-reported and caregiver-reported HRQoL. It remains to be seen whether these results in the normal range for people with a rare disease will change in the coming years. Respondents have had to live with the disease at a time when there were not such dynamic development, extensive research, and associated scientific attention as there are now. The high ratings in the self-reports of the dimension of “Self-Perception” show how confident and satisfied the children/adolescents feel with themselves and their appearance. This dimension also reflects the value of how positively others value him/her. These results can possibly be explained by the fact that in more than half of the interviews (12/23) it is emphasized that more self-efficacy and development opportunities come into focus. “Personally, I wouldn’t use the term [disease] like that. I think I would also explain it like that later, when she can have a little understanding of how a body is formed. Then I would say that some part is broken and we repair it with this injection and everything is normal for them. And the term disease doesn't have to come up at all” (032_002(1), 27.13; Table 2). The feeling that the new therapy is working on the cause rather than just the symptoms seems to be a big contributor to the average HRQoL score. One’s own body is empowered by the new therapy to retain the phosphate; and thus, there is no need to supply the phosphate from the outside. This seems to have a notable effect on the HRQoL assessment.

The dimension “Autonomy” is the only dimension in our study that was rated below the German reference values but above international reference values by both children and adolescents (52.76 ± 9.86) and caregivers (52.23 ± 8.19). But in the qualitative interviews they present examples of a gain in autonomy: “Everyday examples now are simply sleepovers at friends’ houses. We did that before, but then it was always highly complicated because we had to coordinate it with the phosphate administration. And now she can simply do it. And now, for the first time, I have asked her if she would like to go to a holiday camp. I avoided that before because I knew it would be too complicated. [...] I asked her for the first time, I wouldn’t have done that before” (004_004(2), 15.41; Table 2). In the “Autonomy” dimension, improvements in HRQoL are reported. Spontaneous activities, overnight stays with friends, and participation in vacation camps are also possible due to the longer-acting injection, without extensive medication planning. On the other hand, there continue to be limitations in school sports, longer hikes, and heavier exercise reported by nearly all interviewees (22/23), suggesting that this could be the reason for the lower rating.

Our study has limitations. In total, 134 out of around 200 pediatric XLH patients are included in our national registry. However, only 63 out of 100 children participating in this registry and fulfilling the inclusion criterion, i.e., age between 8 and 18 years, participated in our study. Therefore, we cannot exclude that only the most motivated and thus those with better HRQoL participated in the present study which may have biased the study results. We aim to assess HRQoL in the vast majority of XLH patients participating in our multicenter study to exclude such bias in our next analysis. Secondly, we could not directly address the impact of burosumab versus conventional treatment in this analysis as the number of patients on the latter treatment was too small to run a meaningful statistical analysis. Likewise, the good HRQoL scores could be influenced by the subjective experiences of patients transitioning from conventional to burosumab treatment. However, the number of patients primarily started on burosumab was too low to rule out such bias. Finally, the “Financial Resources” were assessed by both children and adolescents and by caregivers with slightly higher *T*-values compared to the reference population. Here, a positive selection cannot be ruled out due to a varied willingness to participate in the survey.

## Conclusions

In this real-world study, overall HRQoL appeared to be average in pediatric XLH patients participating in this German study compared to the general population, which may at least be partly due to the treatment with burosumab which was commenced in the vast majority of patients and associated with improved physical health and well-being in the qualitative interviews. The results of our study may have implications for clinical practice as they support the concept of primary burosumab treatment in pediatric XLH patients.

## Supplementary Information

Below is the link to the electronic supplementary material.

Graphical abstract (PPTX 81.0 KB)

## Data Availability

Datasets are available on request to the corresponding author.
