# Supplementary material for: Health-related quality of life of children with X-linked hypophosphatemia in Germany
Source: Pediatr Nephrol. 2024 Jun 25;39(11):3221–31. doi: 10.1007/s00467-024-06427-0 (PMC11413074; doi:10.1007/s00467-024-06427-0)
Supplement: Supplementary file 1 — Graphical abstract (PPTX 81.0 KB) [file 467_2024_6427_MOESM1_ESM.pptx]

## Slide 1
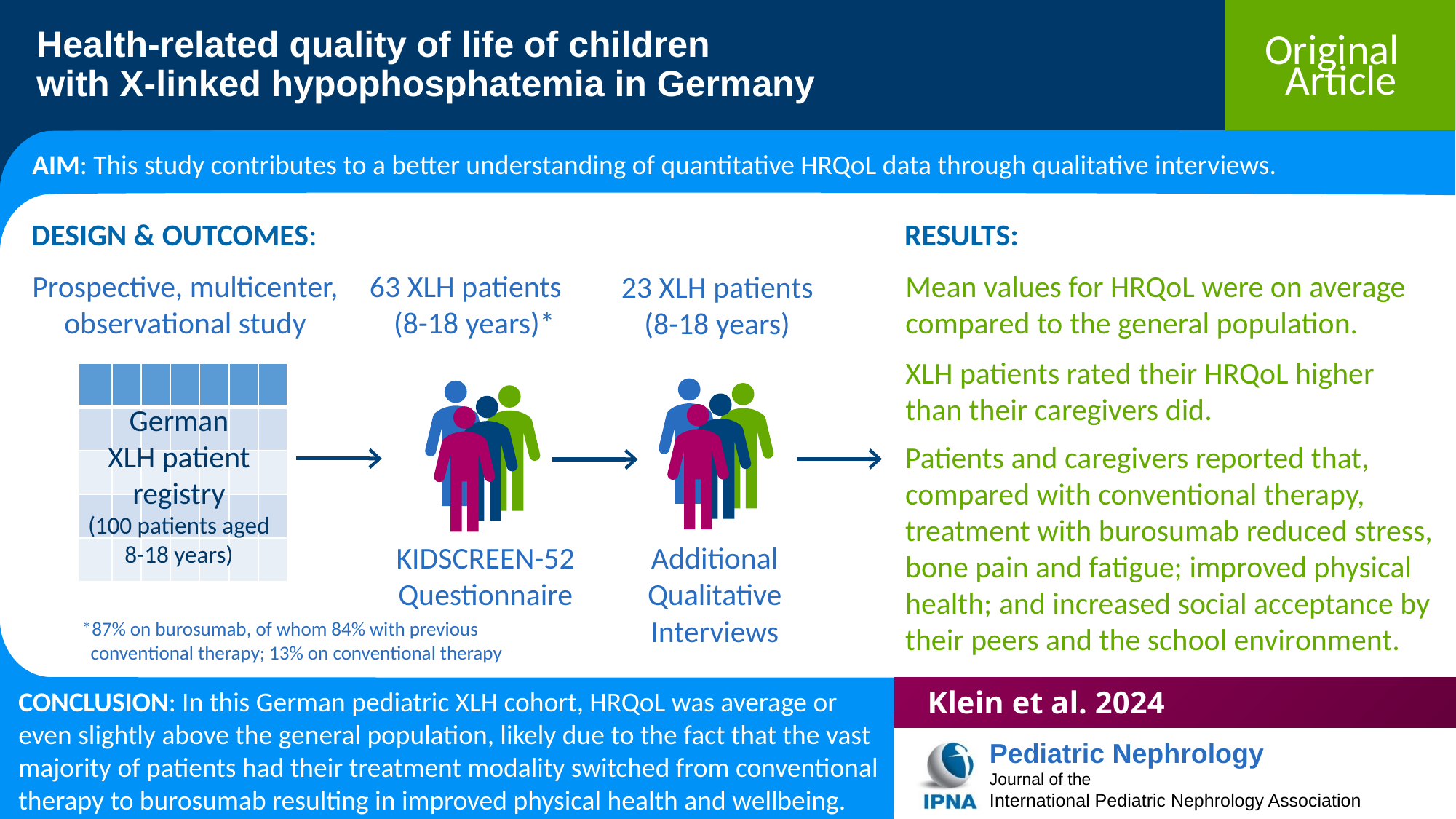

Health-related quality of life of children
with X-linked hypophosphatemia in Germany
AIM: This study contributes to a better understanding of quantitative HRQoL data through qualitative interviews.
DESIGN & OUTCOMES:						RESULTS:
Prospective, multicenter, observational study
63 XLH patients
(8-18 years)*
Mean values for HRQoL were on average compared to the general population.
XLH patients rated their HRQoL higher than their caregivers did.
Patients and caregivers reported that, compared with conventional therapy, treatment with burosumab reduced stress, bone pain and fatigue; improved physical health; and increased social acceptance by their peers and the school environment.
23 XLH patients
(8-18 years)
| | | | | | | |
| --- | --- | --- | --- | --- | --- | --- |
| | | | | | | |
| | | | | | | |
| | | | | | | |
| | | | | | | |
German
XLH patient registry
(100 patients aged 8-18 years)
Additional Qualitative Interviews
KIDSCREEN-52 Questionnaire
*87% on burosumab, of whom 84% with previous
 conventional therapy; 13% on conventional therapy
Klein et al. 2024
CONCLUSION: In this German pediatric XLH cohort, HRQoL was average or even slightly above the general population, likely due to the fact that the vast majority of patients had their treatment modality switched from conventional therapy to burosumab resulting in improved physical health and wellbeing.
